# Evaluating Telehealth Implementation in the Context of Pediatric Chronic Pain Treatment during COVID-19

**DOI:** 10.3390/children8090764

**Published:** 2021-08-31

**Authors:** Patricia A. Richardson, Delana M. Parker, Krystal Chavez, Kathryn A. Birnie, Elliot J. Krane, Laura E. Simons, Natoshia R. Cunningham, Rashmi P. Bhandari

**Affiliations:** 1 Departments of Pediatric Psychology and Pediatric Pain and Palliative Medicine, Helen DeVos Children’s Hospital, Grand Rapids, MI 49503, USA; 2 Department of Pediatrics and Human Development, Michigan State University College of Human Medicine, East Lansing, MI 48824, USA; 3 Department of Psychiatry, Dell Medical School and Dell Children’s Medical Center, University of Texas at Austin, Austin, TX 78712, USA; delanamparker@gmail.com; 4 Department of Digital Health, Stanford Children’s Health, Palo Alto, CA 94304, USA; krchavez@stanfordchildrens.org; 5 Department of Anesthesiology, Perioperative and Pain Medicine, University of Calgary, Calgary, AB T2N 1N4, Canada; kathryn.birnie@ucalgary.ca; 6 Department of Anesthesiology, Perioperative, and Pain Medicine, Stanford University School of Medicine and Stanford Children’s Health, Stanford, CA 94305, USA; ejkrane@stanford.edu (E.J.K.); lesimons@stanford.edu (L.E.S.); rbhandari@stanford.edu (R.P.B.); 7 Department of Family Medicine, Michigan State University College of Human Medicine, East Lansing, MI 48824, USA; natoshia@msu.edu

**Keywords:** telehealth, pediatric pain, specialty care, implementation science, COVID-19

## Abstract

Telehealth has emerged as a promising healthcare delivery modality due to its ability to ameliorate traditional access-level barriers to treatment. In response to the onset of the novel coronavirus (COVID-19) pandemic, multidisciplinary pain clinics either rapidly built telehealth infrastructure from the ground up or ramped up existing services. As the use of telehealth increases, it is critical to develop data collection frameworks that guide implementation. This applied review provides a theoretically-based approach to capitalize on existing data sources and collect novel data to inform virtually delivered care in the context of pediatric pain care. Reviewed multisource data are (1) healthcare administrative data; (2) electronic chart review; (3) clinical health registries; and (4) stakeholder feedback. Preliminary telehealth data from an interdisciplinary pediatric chronic pain management clinic (PPMC) serving youth ages 8–17 years are presented to illustrate how relevant implementation outcomes can be extracted from multisource data. Multiple implementation outcomes were assessed, including telehealth adoption rates, patient clinical symptoms, and mixed-method patient-report telehealth satisfaction. This manuscript provides an applied roadmap to leverage existing data sources and incorporate stakeholder feedback to guide the implementation of telehealth in pediatric chronic pain settings through and beyond COVID-19. Strengths and limitations of the modeled data collection approach are discussed within the broader context of implementation science.

## 1. Introduction

In the early weeks of the COVID-19 pandemic, virtually-delivered care transitioned from being an innovative service delivery method to an essential healthcare platform. Providing healthcare using telehealth enabled pediatric chronic pain clinics to increase patient and staff safety while ensuring continuity of care [1,2]. The expansion of telehealth was further supported by state and federal policy changes, including the Medicaid telehealth expansion, that lifted telehealth restrictions and established parity in reimbursement rates for virtual vs. in-person care. Assuming these policy changes persist, it is likely that standards of healthcare, including chronic pain treatment, will incorporate virtual intervention in the long term [3,4]. The goal of this applied review is to provide a resourceful and theoretically informed perspective on how to assess telehealth implementation by leveraging existing data sources and collecting stakeholder feedback. First, data sources and associated implementation outcomes are reviewed. To model this approach, a case example of telehealth implementation from a multidisciplinary pediatric chronic pain clinic is presented and discussed as it relates to the broader implementation science literature. 

As with any innovative service delivery modality, there is a need for healthcare systems to develop ongoing data collection frameworks that evaluate and guide implementation over time. Such efforts are essential to ensure successful integration at both patient care and systems levels. These data can also be leveraged to quantify cost savings of telehealth programs, despite substantial financial and time investment required by healthcare systems at the outset. Implementation effectiveness, a scientific method that allows for study of the delivery method process, may be useful for understanding data collection frameworks that support telehealth for pediatric pain treatment. For example, traditional in-person cognitive behavioral therapy (CBT) is an evidence-based approach that has been found to improve pain and associated functional disability among pediatric patients with chronic pain. More recent findings have shown that CBT for pain can also be delivered effectively using online (eHealth) modalities (i.e., implementation effectiveness) [5,6,7]. Implementation effectiveness is inventoried using implementation outcomes, characterized by Proctor and colleagues as acceptability, feasibility, and sustainability, among others [8]. See Table 1 for Proctor’s eight implementation outcomes with associated definitions. 

Overarching guidance for developing telehealth research programs has been proposed by Fatehi and colleagues in the Five Stage Model for Comprehensive Research on Telehealth [9]. The authors identified the following stages of development: (1) concept development; (2) service design; (3) pre-implementation; (4) implementation; and (5) post-implementation. Ideally, clinicians, researchers, and healthcare systems would develop thoughtful, theory-driven plans for the ongoing assessment of telehealth implementation as programs evolve over time. However, due to COVID-19, many chronic pain programs have been thrust into the latter two stages of this model, as urgent time constraints required the rapid development or expansion of existing telehealth services for immediate deployment. In this context, practical, applied guidance for assessing implementation outcomes is needed.

This applied review seeks to draw from Proctor’s [8] foundational implementation science evaluation framework to provide guidance on how: (1) clinicians can capitalize on existing multisource data to guide telehealth implementation; and (2) establish novel data sources to assess stakeholder feedback. The focus of this review will be specific to pediatric chronic pain management, though likely has broader applications. Although the Covid-19 pandemic has necessitated a rapid execution of telehealth implementation of clinical care and research for many, it has also accelerated the field’s ability to disseminate specialized care by removing some traditional access-level barriers to treatment. Ultimately, an applied and patient-centered roadmap is needed to shape the successful delivery of telehealth services through and beyond COVID-19. Literature for this applied review was identified through a non-systematic search in PubMed and Google Scholar utilizing the following search terms: child, pediatric, chronic pain, healthcare administrative data, chart review, electronic health record, registry, stakeholder. No language or other restrictions were applied. We also searched reference lists of papers that were identified as particularly relevant. 

### 1.1. Identifying Multisource Data

There is a range of multisource data that can be leveraged to examine telehealth implementation outcomes. These sources may include (1) healthcare administrative data; (2) chart review; (3) clinical registries; and (4) mixed-method patient and provider feedback, among others. Many of these sources are readily available within most healthcare systems (e.g., healthcare administrative data, chart review), whereas others may require new methods of data collection or tailoring of existing quality improvement or service feedback methods (e.g., mixed-method patient and provider feedback). What follows is a brief review of each data source and examples of associated implementation outcomes. Table 2 presents a summary of implementation outcomes that can be extracted from each source of data.

#### 1.1.1. Healthcare Administrative Data 

Broadly, healthcare administrative data (HAD) refers to the routine collection of diagnostic codes, procedure codes, drug prescriptions, managed care claims, and patient demographic information [10,11]. Although HAD is obtained for administrative and billing purposes, these large-scale data also provide a valuable window into service utilization that is able to be stratified by patient demographics and disease status. HAD can be employed to examine the real-world impact of cultural and systems-level changes on healthcare and shed light on social inequalities that exist within healthcare [12]. Along with these incredible strengths, there are also challenges of HAD to consider. Given that HAD are typically not collected systematically with the intention of being utilized as research data, HAD requires special ethical and legal considerations to protect patient confidentiality, as well as time-intensive restructuring and recoding of data to be usable for statistical analyses [12,13]. See Connelly and colleagues [12] and Parsons and colleagues [13] for expanded reviews outlining the use of HAD to advance social science. Mahrer et al. demonstrated how HAD can be leveraged to conduct cost analyses of interdisciplinary chronic pain treatment [14]. 

#### 1.1.2. Electronic Health Record

Retrospective chart review via electronic health record (EHR) can provide insight into an individual patient’s demographic/geographic factors, service utilization, diagnoses, treatment characteristics, and trajectory of symptoms over time, among other indices [15,16,17]. EHRs also afford an opportunity to gather information about service implementation within a service system or group of providers. For example, it is possible to compare rates of telehealth use among providers within a group to assess whether adoption has been embraced by providers equally, as well as rates of provider appointment slot utilization and patient no-shows. Within the chronic pain literature, screening tools within EHR have been successfully implemented to identify patients with anxiety and functional disability among youth with abdominal pain [18]. This approach to screening has multiple implications for use with telehealth, including screening for patient appropriateness for telehealth-delivered chronic pain treatment. Vassar and Holzmann review EHR methodological considerations to support the extraction of valid and reliable data [19].

#### 1.1.3. Clinical Health Registries 

Clinical health registries capture information about patient symptom presentation and severity based on empirically validated and normed self- and proxy- (caregiver) report measures. Registries are aligned with the goals of measurement-based care (MBC) that seek to harness patient-reported outcomes (PROs) to inform and improve clinical decision-making [20]. Clinical registries and the evaluation of PROs may be particularly relevant for certain disease presentations, including chronic pain. For pediatric patients with chronic pain, symptom etiology is often unclear, and in the absence of observable disease markers, clinical decision making often relies on validated patient- and proxy-report data that capture pain experience. Common domains assessed include pain intensity, functional impairment, and mental health. Data extracted from clinical registries can be used to inform patient appropriateness for telehealth. For example, patients with higher mobility challenges or those traveling a great distance for care may be particularly amenable to telehealth, while patients endorsing elevated risk status (e.g., suicidal ideation) may be more appropriate for in-person care. Furthermore, there are commonly used empirically validated measures across chronic pain programs, such as the National Institutes of Health Patient-Reported Outcomes Measurement Information System (PROMIS) [21]. Consistency in measurement across sites supports cross-site collaborations and comparisons. 

One clinical pediatric chronic pain registry is the Pediatric Collaborative Health Outcomes Registry (Peds-CHOIR) at Stanford University [22]. Peds-CHOIR is an open-source clinical registry with flexible web-based interface and graphical capabilities to inform point of care decisions and generate patient-oriented research [23,24,25,26,27,28,29]. Peds-CHOIR incorporates classical testing theory-based measures and item-response theory measures administered via computer adaptive testing, including PROMIS [21]. 

#### 1.1.4. Mixed Method Stakeholder Feedback

The NIH and Patient-Centered Outcomes Research Institute (pcori) defines stakeholders as a “broad range of communities [that] have a stake in generating useful and relevant healthcare research evidence” and may include patients, caregivers, clinicians, community members, health care purchasers, payers, industry, hospitals and other health systems, policy-makers, training institutions, and researchers [30]. Obtaining stakeholder input into program development is essential, as patient feedback generates services patients are more likely to use and find helpful, while provider perceptions may influence their likelihood of adopting approaches into their practice [31,32]. Multiple research programs, including Community-Based Participatory Research (CBPR), Participatory Action Research (PAR), and Patient-Engaged Research (PER) provide frameworks for how researchers and patients collaborate as co-investigators to generate and answer research questions [33,34,35]. Common methods of obtaining stakeholder feedback include satisfaction surveys and focus groups. Feedback is ideally obtained using a mixed-method approach, which involves obtaining quantitative (e.g., ratings on satisfaction questions) and qualitative data (e.g., responses to open-ended questions). Mixed-method feedback grants a rich perspective on the strengths and limitations of telehealth and may be better able to address the complexities of implementation as compared to exclusive use of either quantitative or qualitative feedback [36].

### 1.2. Applied Case Example: Findings from a Pediatric Chronic Pain Clinic

Preliminary data are presented from a tertiary pediatric chronic pain management clinic (PPMC) located in northern California from 2018 to March 2020 to illustrate how several relevant implementation outcomes can be extracted from multisource data. Data and associated analyses were not used to test a priori hypotheses or comment on the efficacy or feasibility of telehealth. Participants were children ages 8–17 years who presented for interdisciplinary specialty care treatment at the PPMC. Telehealth implementation outcomes, as guided by Proctor and colleagues [8], were obtained from the following three data sources (duration of data collection): (1) chart review via EHR (January 2018 to April 2020); (2) a clinical health registry, Peds-CHOIR (January 2018 to December 2019); and (3) mixed-method patient-report service satisfaction (2018 to 2019). Implementation outcomes will be italicized and in parentheses throughout the result. This study was approved as a retrospective chart review by the University’s Institutional Review Board and all data were collected a part of routine clinical care. 

### 1.3. Procedure

Standard, in-person interdisciplinary care at the PPMC often includes weekly follow-up with a pain psychologist and physical therapist and monthly follow-up with a pain physician and/or nurse practitioner. More intensive programming (i.e., daily treatment) is available for patients with higher functional decline and greater treatment need at intake. 

Telehealth rollout across the hospital system, including the PPMC, was managed by the hospital digital health team. The digital health team consisted of four experienced ambulatory operations team members who worked in close collaboration with information services (IS) liaisons. The hospital telehealth implementation framework was based on published guidelines from the American Medical Association, American Academy of Pediatrics and American Telemedicine Association [37,38,39]. At the outset, telehealth services were primarily clinic-to-home (provider in clinic and patient at home). Secondary to the COVID-19 panic, home-to-home (provider at home and patient at home) telehealth was instituted. In order for a provider to be eligible for home-to-home telehealth, each provider, while at home, scheduled a virtual visit with a member of the digital health team. During this visit, the digital health team member assessed provider home internet security and bandwidth ensured that provider devices were encrypted to hospital standards, and conducted sound/video/microphone checks. The digital health team also provided guidance for optimal environmental aspects of telehealth visits: visits were to be conducted in a private space, with a neutral backdrop, out of direct sunlight, and in a position where the camera was approximately eye-level. 

The PPMC began offering virtually-delivered care in January 2018 by training two providers (one pain psychologist and one pain physician). Patients were eligible for telehealth when they had no acute safety concerns or severe psychopathology, as determined by treating providers. Virtual visits were conducted using digital infrastructure offered through the institution’s EHR (EPIC) or Zoom (https:zoom.us, via a secure, end-to-end encrypted and HIPAA-compliant university server). Administrative staff were trained how to register, schedule, and check-in patients (verify physical address, presence of caregiver in case of safety/medical concern, and problem-solve connectivity issues), and collect co-pays for telehealth visits. 

With regard to availability of multisource data at the PPMC, EHR and Peds-CHOIR were extant data collection systems prior to the introduction and rapid transition to telehealth. EHR data were extracted for this study by a trained data analyst through the hospital’s research informatics department. As a part of standard clinical care, all patients completed Peds-CHOIR within 7 days preceding initial interdisciplinary clinic intake and at monthly intervals. Questionnaires were completed electronically by patients through a secure URL link emailed to them upon registering for their clinic appointment. 

Patient-reported telehealth satisfaction surveys were developed and collected by the digital health team to evaluate telehealth across the hospital system. After completing a telehealth visit, all patients were automatically emailed optional satisfaction surveys via Typeform, an anonymous survey platform. Surveys were distributed to patients’ primary email address on file, which was most commonly the caregiver email address. Surveys included items rated on a Likert scale and open-ended questions, see Table 4 for survey items. 

### 1.4. Analytic Plan

Analyses were primarily descriptive in nature (e.g., mean, standard deviation, frequency). Independent sample t-tests were used to examine differences on patient-reported pain and function among patients who received combination in-person and telehealth care versus patients receiving only in-person care. All analyses were conducted using SPSS 25 (IBM SPSS, 2017) [40].

## 2. Results

### 2.1. EHR

**Figure 1:**
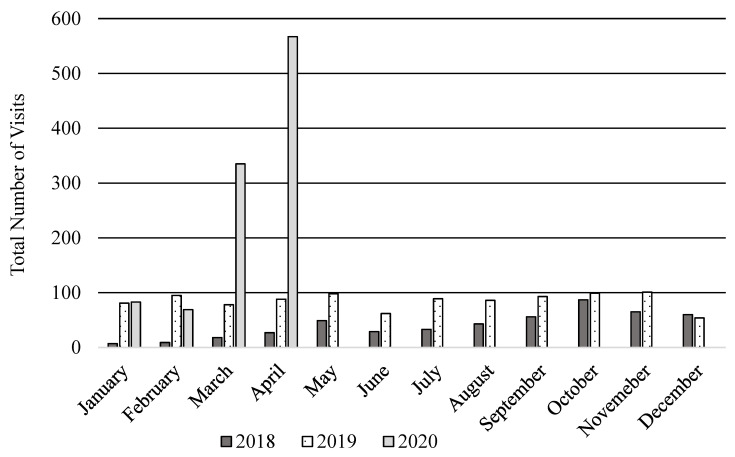
Frequency of PPMC telehealth visits from 2018 to 2020.

Adoption and penetration of telehealth was examined by charting use of telehealth across clinic providers and summing the total number of PPMC telehealth visits from 2018 to March 2020. The telehealth program started in 2018 with 1 pain psychologist and 1 pain physician offering care via telehealth. By the end of 2018, three psychologists and 1 physician integrated telehealth into their practice. In 2019, 6 psychologists, 6 physicians, and 2 nurse practitioners offered care via telehealth, and by 2020 all providers utilized telehealth (7 psychologists, 6 physicians, 2 nurse practitioners). The average number of PPMC monthly telehealth visits increased from 40 visits (2018) to 85 visits (2019) to 263 visits (2020). At the start of 2020 (prior to the onset of the COVID-19 pandemic), approximately 30% of mental health and 10% of medical follow-ups at the PPMC were conducted via telehealth. In response to the COVID-19 pandemic, by the second week of March 2020, 100% of all patient visits were converted to home-to-home telehealth visits. See Figure 1 for PPMC telehealth visits across years 2018 to 2020.

In 2018, psychology visits accounted for 92% of PPMC telehealth. In 2020, psychology accounted for 73% of visits, as physicians and nurse practitioners increased telehealth use secondary to COVID-19. EHR was also employed to compare PMMC rates of patient cancelations and no-shows across 2018 and 2019 (sustainability). When considering all PPMC visits (telehealth and in-clinic), clinic rates of no-shows were stable (2018: 7.1%; 2019: 7.4%), while patient-initiated cancellations decreased (2018: 12.74%; 2019: 10.69%).

Across the overall PPMC sample (*N* = 587) from 2018 to 2019, 19.8% (*n* = 116) of patients engaged in at least one follow-up session via telehealth with an interdisciplinary provider (acceptability). Among telehealth users, patients had a range of 1–37 telehealth visits and engaged in just over four telehealth visits, on average (*M* = 4.23, *SD* = 5.77). Patient zip code was extracted from the EHR and used to generate estimates of time savings. PPMC families saved an average of 2.74 h of drive time per visit by attending visits via telehealth as opposed to in-person care. 

### 2.2. Clinical Health Registry

The PPMC clinical health registry, Peds-CHOIR, captured information about patient pain experience and clinical symptoms. This applied review presents Peds-CHOIR data from January 2018 to December 2019. Patients were predominantly female (71.6%). Self-identified race was: White or Caucasian (51.4%), Biracial/Other (30.2%), Asian (10.1%), Black or African American (2.2%), American Indian or Alaskan (0.2%), and Native Hawaiian/Pacific Islander (0.5%); 5.5% declined to report race. Primary pain concerns per evaluating clinician were reported as: headache (33%), musculoskeletal pain (18%), abdominal pain (15%), complex regional pain syndrome (10%), fibromyalgia/pain amplification syndrome (5%), joint pain (4%), and other (15% e.g., jaw pain).

Patients were stratified into a “combined telehealth and in-person” group if they participated in at least one visit via telehealth (many also attended in-person visits). Patients in the “in-person only” group attended only in-clinic follow-up appointments. Based on PROMIS clinical thresholds [41], in the combined group, patient-reported mobility, pain interference, and fatigue were considered to be in the moderately clinically elevated range. Peer relations, anxiety, and depressive symptoms were within normal limits. When comparing the combined group to the in-person only group at initial intake (appropriateness), the combined group had higher report of symptoms across all assessed domains. Independent sample t-tests at baseline revealed that the combination group reported significantly higher pain intensity (t(585) = −2.72, *p* = 0.007) and pain interference (t(585) = −2.23, *p* = 0.02) as compared to in-person only (Table 3). In addition to intake data, patients completed Peds-CHOIR at multiple scheduled follow-ups over the course of treatment, including 1-, 3-, and 6-months post-treatment initiation. These data could be analyzed to examine patient symptom trajectories over the course of treatment (feasibility, sustainability). 

### 2.3. Mixed-Method Stakeholder Feedback

Among PPMC patients who attended at least one telehealth appointment (*n* = 116), a subset of patients (*n* = 57) reported on satisfaction during the years of 2018 and 2019. Although stakeholders rated each item high on a Likert scale, there were technical issues that occurred at the beginning of telehealth rollout, that may have been better captured by qualitative feedback (e.g., “The sound cut out a few times and at one point we were cut off completely”). Based on qualitative feedback in survey version 1 (2018), it was clear that technical issues were a key area for improvement (sustainability). The digital health team used this feedback to adjust the digital platform and create survey version 2 (2019), in order to better capture technical function during telehealth visits. Qualitative data also helped the digital health team to recognize the importance of generating patient links to virtual sessions could support multiple providers and/or family members connecting from different devices. This improvement fostered provider ability to conduct family sessions and care conferences, while reducing child and caregiver need to miss school/work for appointments. See Table 4 for a summary of survey items and ratings by PPMC patients (primarily caregivers) who completed the telehealth satisfaction survey (acceptability, satisfaction).

## 3. Discussion

Within a span of weeks following the onset of COVID-19, virtually-delivered care shifted from being a promising service delivery method to an essential medium for providers to interface with patients. Telehealth enabled the pediatric pain management community to increase staff, patient, and family safety while continuing to provide needed interventions during the pandemic [42]. Beyond its incredible role in facilitating continuity of care during this crisis, telehealth has the potential to ameliorate access-level treatment barriers and resultant healthcare disparities with which our field has contended for decades. As providers and healthcare systems have worked to rapidly respond to the widespread need for virtual care, little time has been afforded to follow guidance from established theoretically-based evaluation models of implementation outcomes [9]. There is a need to establish that virtually-delivered care is feasible and sustainable, well received by key stakeholders, and worthy of ongoing support. Thus, the overarching goal of this applied review was to provide a resourceful and theoretically informed perspective on how clinicians and researchers can evaluate their approach to telehealth implementation. 

Innovative service delivery modalities necessitate data collection frameworks that evaluate and shape service implementation within a particular setting over time, often referred to as implementation science. In their foundational work, Proctor and colleagues characterized eight key outcomes to guide implementation, including acceptability, adoption, appropriateness, cost, feasibility, fidelity, penetration, and sustainability [8]. The present manuscript reviewed the following multisource data that can be harnessed to extract implementation outcomes: (1) healthcare administrative data (HAD); (2) chart review via electronic health records (EHR); (3) clinical health registries; and (4) mixed-method stakeholder feedback. Examples of applied implementation outcomes derived from each data source are summarized in Table 2. While many healthcare systems have access to one or more of these sources, it is important to note that the data sources and implementation outcomes described in this manuscript are not considered to be an exhaustive inventory. It is important to identify sources of data that are unique to one’s institution and assess outcomes that are relevant to a given program’s implementation goals. 

### 3.1. Applications within Tertiary Pediatric Specialty Care

Descriptive data were presented from a tertiary pediatric chronic pain management clinic (PPMC) to demonstrate how implementation outcomes could be extracted from multisource data. Implementation outcomes were obtained from the following three sources: (1) chart review using EHR; (2) a clinical health registry, the Pediatric Collaborative Health Outcomes Registry (Peds-CHOIR) [22]; and (3) mixed-method patient-reported satisfaction. Practically, PPMC implementation outcomes are routinely extracted and reviewed internally to enhance the institution’s telehealth programming. 

#### 3.1.1. Chart Review Using EHR

Chart review via EHR yields data that may include patient demographic/geographic factors, service utilization, diagnoses, and treatment characteristics, among other key data points [15,19]. At the PPMC, chart review was used to obtain the number of telehealth visits over time and by provider, with the goal of assessing adoption and penetration. As shown in Figure 1, the number of patient visits conducted via telehealth increased during 2018 to 2019, and rapidly increased in 2020 when all visits transitioned to telehealth, secondary to COVID-19. Within the current sample, psychologists conducted more sessions via telehealth as compared to other PPMC providers (i.e., physicians and nurse practitioners). This finding is likely related to the practice convention that psychologists generally see patients on a more frequent basis (i.e., weekly) than physicians and nurse practitioners. The nature of behavioral health interventions is also often more easily translated to virtual environment (e.g., minimal need for in-person physical exam). Notably, managed care reimbursement likely also had a bearing on both adoption and penetration. Visit reimbursement across insurance companies gradually increased from 2018 to 2020, when all insurance providers reimbursed for telehealth visits. Insurance-related complexities continue to be a challenge when out-of-state patients seek care at the PPMC, though multiple states and licensing boards are advocating for interjurisdictional practice (e.g., Psychological Interjurisdictional Compact, PSYPACT) [43].

#### 3.1.2. Clinical Health Registry

Clinical health registries are the foundation of measurement-based care and routine outcome monitoring in healthcare. Utilizing valid and normed-measures, registries are able to speak to patient symptom clinical presentation and response to treatment. Self-reported symptom data are often highly relevant for chronic pain diagnoses, as such diagnoses often lack observable disease markers and rely on patient report to guide treatment. With regard to evaluation of implementation, health registries generate metrics that can be used to measure appropriateness, feasibility, penetration of telehealth, among other key indices. Registry data from the PPMC indicated that patients who had at least 1 visit via telehealth had significantly higher levels of pain intensity and pain interference, as compared to patients who only completed in-person visits. Such within-clinic findings may help to elucidate potentially meaningful areas for further inquiry. Routinely collecting clinical registry data is well-aligned with the increasing call for precision medicine practices in pediatric pain [44], and healthcare [45], more broadly, which process patient data (e.g., genetics, disease markers, lifestyle, and psychosocial indices) using healthcare analytics to classify patients into subgroups that guide evidence-based and targeted intervention. 

#### 3.1.3. Mixed-Method Stakeholder Feedback

Incorporating stakeholder input into program development is essential in order to generate services that patients are willing to use and experience as helpful. At the PPMC, the digital health team developed a brief feedback questionnaire. Though the mixed-method patient feedback questionnaire utilized by the PPMC was beneficial to program development, there are multiple ways to strengthen the collection of stakeholder feedback. First, satisfaction questionnaires were completed by a convenience sample of patients which limits generalizability of results and may not reflect the full breadth of user experience. Furthermore, questionnaire items were developed by the digital health team. Utilizing a validated and normed patient satisfaction questionnaire would have been ideal in order to facilitate the comparison of results across time and samples [46]. Evidence-based implementation measurement is a growing field in need of additional work within both telehealth and pediatric populations. As noted in the method, questionnaires were most commonly delivered to the email addresses of caregivers, and thus, were most likely completed by caregivers. Although caregiver feedback is important, it would also be beneficial to develop a system to obtain direct feedback from pediatric patients. With regard to other key stakeholders, provider feedback was not systematically assessed. Informal exchange occurred on a regular basis between providers and the digital health support team at the institution, which facilitated program development. However, without systematic measurement, the ability to statistically report on this iterative process is limited. See Palinkas and Cooper [47] for a broader review of mixed-method stakeholder feedback within the context of implementation science.

### 3.2. Opportunity for Innovation and Ongoing Priorities

Substantive literature has confirmed that optimal chronic pain treatment hinges on multidisciplinary care that may include psychologists, physicians, social workers, physical and occupational therapists, among others [48]. Even prior to the onset of the pandemic, families have faced barriers in accessing these services as widespread availability of interdisciplinary chronic pain treatment is undermined by a shortage of services outside of university centers, significant treatment-related costs, and long provider waitlists [49,50]. Barriers to treatment have contributed to healthcare disparities and insufficient treatment of pain conditions in pediatrics. Harnessing the strengths of digital health technology may, in part, be primed to address current limitations of the field. Although multiple research priorities remain with regard to the integration of evidence-based chronic pain treatment and digital health technology [44,51], it is clear that one largescale impact of the pandemic on healthcare has been a worldwide expansion of telehealth. This rapid growth inherently generates opportunity to advance the science of multidisciplinary virtual care that transcends the COVID-19 crisis. 

The amenability of multidisciplinary care to telehealth delivery relates to type of service provided. For example, most essential components of evidence-based behavioral health treatments translate well to telehealth; freely available internet and smartphone application-based tools can be employed to enhance the telehealth experience. There is greater complexity in delivering other key components of multidisciplinary care in a virtual landscape. Physicians, nurses, and other allied healthcare professionals often rely on physical exam for diagnosis and to evaluate response to treatment. At the outset of the pandemic, when all clinical services at the PPMC were being delivered remotely, physicians and nurse practitioners harnessed their creativity to conduct aspects of physical exams virtually. For example, virtual physical exams included observing muscle bulk symmetry, limb range of motion, gait, upper and lower extremity strength, spine mobility, and enlisting caregivers to assist with limb positioning, and assessing allodynia (exaggerated pain response from touch). There are many elements of a comprehensive physical examination as well as the need for interventional procedures, that cannot yet be replaced by virtual visits. Although the provision of virtually delivered multidisciplinary specialty care is not yet well-explored in extant literature, this is likely a promising area for further inquiry. 

In addition to developing systems for capturing telehealth implementation outcomes, other key priorities remain important to consider as telehealth becomes increasingly embedded in standard healthcare. For example: research to establish the efficacy of telehealth on clinical outcomes with pediatric patients, impact of telehealth on limits of confidentiality, costs to initiate and continue telehealth programming, technology infrastructure security, optimizing telehealth training for staff (e.g., safety practices, billing, documentation), incorporating and advocating for state and federal policy, attending to potential increases in the “digital divide”, among others [52,53,54,55,56,57].

## 4. Conclusions

Telehealth facilitated continuity of care for pediatric patients with chronic pain across the COVID-19 pandemic. Healthcare systems and the federal and state governments, alike, rapidly made practical and administrative changes that facilitated the feasibility of virtually delivered care. However, for many providers and healthcare systems, the urgent need for an alternative to in-person treatment eliminated the window of time needed to develop thoughtful, theory-driven plans for the ongoing evaluation of telehealth implementation. Implementation data is essential to shape services over time in a way that meets the needs of patients, providers, and health care systems, alike. Beyond ensuring the continuity of treatment throughout this crisis, the proliferation of virtually delivered care provides an opportunity to transform existing paradigms of multidisciplinary care, engendering a lasting impact on our ability to reach underserved patients and families. This manuscript provides an applied, resourceful, and theoretically grounded roadmap to harness readily available multisource data in order to evaluate telehealth implementation, and ultimately, to improve patient care beyond the COVID-19 pandemic.

## Figures and Tables

**Table 1:** Taxonomy of Implementation Outcomes as Defined by Proctor and Colleagues [8].

Implementation Outcome	Definition
Acceptability	perception among implementation stakeholders that a given treatment, service, practice, or innovation is agreeable, palatable, or satisfactory.
Adoption	intention, initial decision, or action to try or employ an innovation or evidence-based practice
Appropriateness	perceived fit, relevance, or compatibility of the innovation or evidence-based practice for a given practice setting, provider, or consumer; and/or perceived fit of the innovation to address a particular issue or problem
Cost	cost impact of an implementation effort
Feasibility	extent to which a new treatment, or an innovation, can be successfully used or carried out within a given agency or setting
Fidelity	degree to which an intervention was implemented as it was prescribed in the original protocol or as it was intended by the program developers
Penetration	integration of a practice within a service setting and its subsystems
Sustainability	extent to which a newly implemented treatment is maintained or institutionalized within a service setting’s ongoing, stable operations

**Table 2:** Sample Operational Definitions of Telehealth Implementation Outcomes by Data Source.

Data Source	Implementation Outcome	Operationalization
Healthcare administrative data	Sustainability	Reimbursement/revenue generated through telehealth services
		Are certain diagnostic codes less likely to be reimbursed via telehealth relative to in person visits?
	Cost	Financial investment required to initiate and maintain telehealth service as compared with delivering care in person; healthcare utilization (are patients continuing to seek in-person treatment with other providers, such as ER visits?)
	Efficiency	Rates of same day encounter closures/completion of documentation for telehealth versus in person visits
Electronic health record	Acceptability	Number of patients who agreed to receive healthcare via telehealth
	Adoption	Percentage of daily patient visits conducted via telehealth over time
	Cost	Patient zip/postal code data may be extracted to estimate saved appointment transportation costs
	Penetration	Assess whether there are differences in telehealth use associated with patient demographics
		Does a previously defined risk group (e.g., home bound, complex medical needs, patients in rural area) use telehealth?
	Efficiency	Does treatment length (total number of sessions) differ for services delivered in person relative to remotely?
	Sustainability	Rate of no-shows by appointment type
Clinical health registry	Appropriateness	Severity/type of clinical symptoms
	Feasibility	Change in quality of life and clinical symptoms over time
	Penetration	Characterize patients being seen via telehealth
Mixed method stakeholder feedback	Acceptability	Patient, provider and staff self-reported willingness to utilize telehealth
	Satisfaction	Patient satisfaction with telehealth treatment as delivered
		Provider satisfaction with telehealth treatment as implemented
	Cost	Elicit average transportation, lost work time saved by patient attending an appointment in person relative to telehealth
	Safety	Assess whether providers felt critical safety concerns could be adequately managed via telehealth
	Sustainability	Frequency of technological challenges that result in lost service delivery time
	Fidelity	Did providers utilize telehealth as originally envisioned and were additional novel applications discovered?

**Table 3:** Comparison of Patients in Telehealth vs. TAU Groups on Symptoms at the Time of Initial Evaluation.

	Combined Telehealth and In-Person	In-Person Only
Age	14.75 (2.28)	14.38 (2.49)
Sex	71.6% female	71.5% female
Pain Intensity +	6.18 (2.26)	5.48 (2.48) **
Mobility	64.32 (7.71)	62.73 (8.07)
Pain Interference ^^^	61.48 (7.51)	59.50 (8.15) *
Peer Relationships ^^^	54.05 (10.16)	53.55 (9.39)
Fatigue ^^^	63.26 (12.42)	60.90 (11.55)
Anxiety ^^^	54.01 (9.74)	53.86 (10.40)
Depression ^^^	55.88 (10.30)	55.36 (10.75)
Pain Catastrophizing	30.33 (10.51)	28.33 (11.15)

Note: combined group (*n* = 116) had at least 1 appointment via telehealth and the in-person group (*n* = 471) received only in-person services. Across measures, high scores indicate less optimal function in that domain. + pain intensity rated on an 11-point numeric rating scale. ^^^ PROMIS measures are T scores with *M* = 50 and *SD* = 10. Significant differences between groups were examined using independent sample t-tests. * = *p* < 0.05, ** = *p* < 0.01.

**Table 4:** Patient Stakeholder Survey Responses.

Patient Survey 1 (2018), *n* = 17		
Item	*M*	*SD*
I was able to connect to my Virtual Visit easily	4.27	1.22
I felt that the Virtual Visit was safe and secure	4.93	0.26
The provider was able to address my/my child’s reason for a visit	4.93	0.26
Patient Survey 2 (2019), *n* = 40		
Please rate audio/video quality	4.57	0.68
Ease of connecting	4.33	1.03
Overall experience	4.69	0.66

Note: all items rated on a Likert scale from 1–5 where higher scores reflect greater endorsement of that item.

## Data Availability

The data presented in this study are from a clinical population and are not publicly available.
